# An agenda for research and action toward diverse and just futures for life on Earth

**DOI:** 10.1111/cobi.13671

**Published:** 2021-03-03

**Authors:** C. Wyborn, J. Montana, N. Kalas, S. Clement, F. Davila, N. Knowles, E. Louder, M. Balan, J. Chambers, L. Christel, T. Forsyth, G. Henderson, S. Izquierdo Tort, M. Lim, M. J. Martinez‐Harms, J. Merçon, E. Nuesiri, L. Pereira, V. Pilbeam, E. Turnhout, S. Wood, M. Ryan

**Affiliations:** ^1^ Luc Hoffmann Institute IUCN Conservation Centre Rue Mauverney 28 Gland 1196 Switzerland; ^2^ Institute for Water Futures, Fenner School of Environment and Society Australian National University Canberra ACT 0200 Australia; ^3^ School of Geography and the Environment University of Oxford South Parks Road Oxford OX1 3QY U.K.; ^4^ Department of Environmental Systems Science ETH Zürich Universitätstrasse 8‐22 Zürich 8092 Switzerland; ^5^ Geography and Planning University of Liverpool Liverpool L69 3BX U.K.; ^6^ Institute for Sustainable Futures University of Technology Sydney 253 Jones Street Ultimo NSW 2007 Australia; ^7^ Department of Geography and Environmental Management University of Waterloo 200 University Ave W Waterloo ON N2L 3G1 Canada; ^8^ School of Geography and Development University of Arizona ENR2 Building, South 4th floor 1064 E. Lowell Street Tucson AZ 85721 U.S.A.; ^9^ The Forest Way No 8, 2nd St, D P Nagar, Kotturpuram Chennai Tamil Nadu 600085 India; ^10^ Forest and Nature Conservation Policy Group Wageningen University P.O. Box 47 Wageningen 6700 AA The Netherlands; ^11^ School of Politics and Government (EPyG) National University of San Martin Avenida 25 de Mayo 1021 San Martín Provincia de Buenos Aires 1650 Argentina; ^12^ Department of International Development London School of Economics and Political Science Houghton Street London WC2A 2AE U.K; ^13^ Harry Ransom Center The University of Texas at Austin P.O. Drawer 7219, 300 W 21st Street Austin TX 78712 U.S.A.; ^14^ Institut des Sciences de la Forêt Tempérée Université du Québec en Outaouais 58 rue Principale Ripon QC J0V 1V0 Canada; ^15^ Natura y Ecosistemas Mexicanos A.C. Plaza San Jacinto 23D, San Ángel, Álvaro Obregón Mexico City 01000 Mexico; ^16^ Centre for Environmental Law, Macquarie Law School Macquarie University 6 First Walk Sydney NSW 2109 Australia; ^17^ Center for Applied Ecology and Sustainability (CAPES) Pontificia Universidad Católica de Chile Santiago, Avd. Libertador Bernardo O'Higgins 340 Santiago Chile; ^18^ Instituto de Investigaciones en Educasión Universidad Veracruzana Paseo 112, Nuevo Jalapa Xalapa‐Enríquez 91193 Mexico; ^19^ Social Science Faculty African Leadership University (ALU) Powder Mill Road Pamplemousses 21001 Mauritius; ^20^ Stockholm Resilience Centre Stockholm University Kräftriket 2B Stockholm SE‐10691 Sweden; ^21^ Copernicus Institute of Sustainable Development Utrecht University Princetonlaan 8a Utrecht 3584 CB The Netherlands; ^22^ Centre for Complex Systems in Transition Stellenbosch University 19 Jonkershoek Rd, Mostertsdrift Stellenbosch 7600 South Africa; ^23^ Clear Horizon Consulting 132B Gwynne St Cremorne VIC 3121 Australia; ^24^ Future Earth 1250 Guy St, Montreal Quebec ON H3H 2L3 Canada

**Keywords:** Anthropocene, biodiversity research, diversity, futures, justice, narratives, transformative change, Antropoceno, cambio transformativo, diversidad, futuro, investigación sobre la biodiversidad, justicia, narrativas

## Abstract

Decades of research and policy interventions on biodiversity have insufficiently addressed the dual issues of biodiversity degradation and social justice. New approaches are therefore needed. We devised a research and action agenda that calls for a collective task of revisiting biodiversity toward the goal of sustaining diverse and just futures for life on Earth. Revisiting biodiversity involves critically reflecting on past and present research, policy, and practice concerning biodiversity to inspire creative thinking about the future. The agenda was developed through a 2‐year dialogue process that involved close to 300 experts from diverse disciplines and locations. This process was informed by social science insights that show biodiversity research and action is underpinned by choices about how problems are conceptualized. Recognizing knowledge, action, and ethics as inseparable, we synthesized a set of principles that help navigate the task of revisiting biodiversity. The agenda articulates 4 thematic areas for future research. First, researchers need to revisit biodiversity narratives by challenging conceptualizations that exclude diversity and entrench the separation of humans, cultures, economies, and societies from nature. Second, researchers should focus on the relationships between the Anthropocene, biodiversity, and culture by considering humanity and biodiversity as tied together in specific contexts. Third, researchers should focus on nature and economies by better accounting for the interacting structures of economic and financial systems as core drivers of biodiversity loss. Finally, researchers should enable transformative biodiversity research and action by reconfiguring relationships between human and nonhuman communities in and through science, policy, and practice. Revisiting biodiversity necessitates a renewed focus on dialogue among biodiversity communities and beyond that critically reflects on the past to channel research and action toward fostering just and diverse futures for human and nonhuman life on Earth.

## Abstract 2

*Article Impact Statement*: Placing diversity and justice at the heart of transformative change for biodiversity offers important new directions for research and action.

## Abstract (es)

Una Agenda para la Investigación y la Acción hacia un Futuro Diverso y Justo para la Vida sobre la Tierra

**Resumen:**

Las décadas de investigación e intervenciones políticas sobre la biodiversidad han tratado significativamente los temas de la degradación de la biodiversidad y la justicia social. Debido a esto, se requieren nuevas estrategias. Diseñamos una agenda de investigación y acción que llama a la labor colectiva de revisar la biodiversidad hacia el objetivo de sustentar un futuro diverso y justo para la vida sobre la Tierra. Cuando se revisa la biodiversidad, se requiere de una reflexión crítica sobre las investigaciones, políticas y prácticas presentes y pasadas sobre la biodiversidad para inspirar un pensamiento creativo acerca del futuro. Desarrollamos la agenda por medio de un proceso de diálogo de dos años que involucró a casi 300 expertos de diversas disciplinas y localidades. Este proceso estuvo orientado por el conocimiento de las ciencias sociales que muestra cómo la investigación y la acción para la biodiversidad están sostenidas por las opciones de cómo están conceptualizados los problemas. Reconocimos al conocimiento, la acción y la ética como inseparables y sintetizamos un conjunto de principios que ayuda a navegar la labor de revisar la biodiversidad. La agenda articula cuatro áreas temáticas para la investigación en el futuro. Primero, los investigadores necesitan revisar las narrativas de la biodiversidad mediante el cuestionamiento de las conceptualizaciones que excluyen a la diversidad y consolidan la separación entre humanos, culturas, economías y sociedades y la naturaleza. Segundo, los investigadores deberían enfocarse en las relaciones entre el antropoceno, la biodiversidad y la cultura al considerar a la humanidad y la biodiversidad como interconectadas en contextos específicos. Tercero, los investigadores deberían enfocarse en la naturaleza y las economías al tener en mejor cuenta la interacción de las estructuras de los sistemas económico y financiero como conductores nucleares de la pérdida de la biodiversidad. Finalmente, los investigadores deberían permitir la investigación y acción transformadoras de la biodiversidad al reconfigurar las relaciones entre las comunidades humanas y no humanas dentro y a través de la ciencia, la política y la práctica. La revisión de la biodiversidad necesita de un enfoque renovado sobre el diálogo entre las comunidades de la biodiversidad y más allá, que reflexione críticamente sobre el pasado para canalizar a la investigación y acción hacia el fomento del futuro justo y diverso para la vida humana y no humana sobre la Tierra.

## Introduction

The multiple challenges undermining relations between people and nature pose a conundrum for research and action. Despite decades of research and policy interventions, the dual issues of biodiversity degradation and social injustices continue apace (Leach et al. 2018; Díaz et al. 2019; IPBES 2019). With this essay, we articulate an agenda for research and action centered on a collective task of revisiting biodiversity toward the goal of sustaining diverse and just futures for life on Earth. Revisiting biodiversity involves critically reflecting on past and present research, policy, and practice concerning biodiversity to inspire creative thinking about the future.

Developed as part of a 2‐year dialogue under the Biodiversity Revisited Initiative, this agenda is intended for a broad community of researchers and practitioners from within academia, government, NGOs, research‐funding organizations, and other institutions and communities. We propose a principle‐based approach to guide how research and action are shaped, conducted, and funded and identify 4 thematic directions for the future. The task of revisiting biodiversity requires ongoing dialogue across disciplines, sectors, knowledge systems, and geographies to ensure participation of an array of voices. This agenda is intended as an initial provocation to stimulate such transdisciplinary dialogue and thereby strengthen the diversity of disciplinary perspectives and collaborations in biodiversity research and action (after Teel et al. 2018).

## Revisiting Biodiversity

The revisiting biodiversity agenda calls for collective action to sustain diverse and just futures for life on Earth. This follows a longstanding legacy of political activism, debate, and social research that has sought to reframe the place of people with respect to biodiversity, for example, community‐based conservation (e.g., Berkes 2004), integrated conservation and development (e.g., Adams et al. 2004), environmental justice (e.g., Agyeman et al. 2016), political ecology (e.g., Escobar 1998), and anthropology (e.g., Sawyer & Agrawal 2000), as well as recent normative calls for more integrated, inclusive, and transformative approaches to biodiversity research and action (e.g., Colloff et al. 2017; Díaz et al. 2019;Editors Nature Ecology & Evolution 2020).

There are many reasons to revisit biodiversity research and action. Despite decades of scholarship, global conservation targets (e.g., CBD 2010; UN 2015), and localized conservation successes (e.g., Conservation Optimism 2020), biodiversity is declining at unprecedented rates (IPBES 2019; CBD 2020) and the systemic drivers of species extinction, habitat destruction and unsustainable resource exploitation, persist (Johnson et al. 2018). Meanwhile, conservation is plagued by its colonial legacy (Sawyer & Agrawal 2000), and the mixed impacts it has on local communities (Naughton‐Treves et al. 2005) demand greater attention to issues of justice (Armstrong 2019), race (Editors Nature Ecology & Evolution 2020), and inequality in biodiversity research and action (Leach et al. 2018). These concerns, and others, are compounded by misaligned incentive structures, short‐term funding cycles, overly simplistic or prescriptive interventions (i.e., Rosenschöld 2019), and the choices that are made in how to look at the problem of biodiversity for research and action (i.e., Rose 2018; Wyborn et al. 2019). The task of revisiting biodiversity therefore requires a collective reflection on the what and how of research, education, and action to draw together diverse perspectives in innovative and inclusive ways.

The agenda seeks to be transformative with respect to the driving goal of sustaining diverse and just futures for life on Earth. In doing so, it broadens the normative goal of biodiversity research and action in line with scholarship in parallel fields, such as sustainability science (Kates et al. 2001), while retaining life on Earth as its unified object of inquiry. Recognizing the interconnections between biological and cultural diversity and the central place people play in shaping biodiversity futures (Rozzi et al. 2018), we seek to extend the long‐held norm of diversity as desirable in biodiversity research (i.e., Soulé 1985) to include humans and their cultures.

The agenda places justice as equal to and inseparable from aspirations to sustain biodiversity. We therefore hope to further a multidimensional view of justice that encompasses the distribution of rights, responsibilities, costs, and benefits of biodiversity interventions (distributive justice), the role and ability of different stakeholders to contribute to decision making (procedural justice), recognition of different histories and identities, human and nonhuman communities (multispecies justice) (Schlosberg 2007; Heise 2016), and the connected agendas of environment, race, class, gender, and social justice (environmental justice) (Agyeman et al. 2016). Justice invokes the moral and legal obligations owed to individuals by societies and their institutions and therefore, more so than, for example, equity, implies both rights and responsibilities (Armstrong 2019). Attention to justice has a longstanding history within sustainable development, environmental justice, and political ecology; however, it deserves greater emphasis across all forms of biodiversity research. Adopting a normative goal that places justice on an equal footing to biodiversity would be both transformative and require transformative change to reconfigure the underlying processes, structures, and outcomes (after Diaz et al. 2019; Scoones et al. 2020) that shape biodiversity research, education, and action.

This agenda builds on other research agendas related to biodiversity (i.e., Sandbrook et al. 2013; Bennett et al. 2017; Mori et al. 2017; Burch et al. 2019; Sutherland et al. 2020), sustainability science (Kates et al. 2001), and environmental governance (Leach et al. 2018; Cumming et al. 2020). Such current approaches to research and action have their strengths and weaknesses (Wyborn et al. 2019). In developing this agenda, we looked across, rather than within, these existing traditions as a means to facilitate a transdisciplinary dialogue. The emergent result is a collective task of revisiting biodiversity with the aim to critically reflect on and renew the objects at the center of a dialogue about research and action. The agenda's niche emerges from a commitment to the boundary object of revisiting biodiversity. Boundary objects are concepts that embody different meanings across cultures, while providing enough commonality to allow different groups to communicate and collaborate (Star & Griesmer 1989). Revisiting biodiversity is proposed as a boundary object and convening device to create arenas where ideas and actions can coevolve.

## An Approach to Revisiting Biodiversity

**Figure 1:**
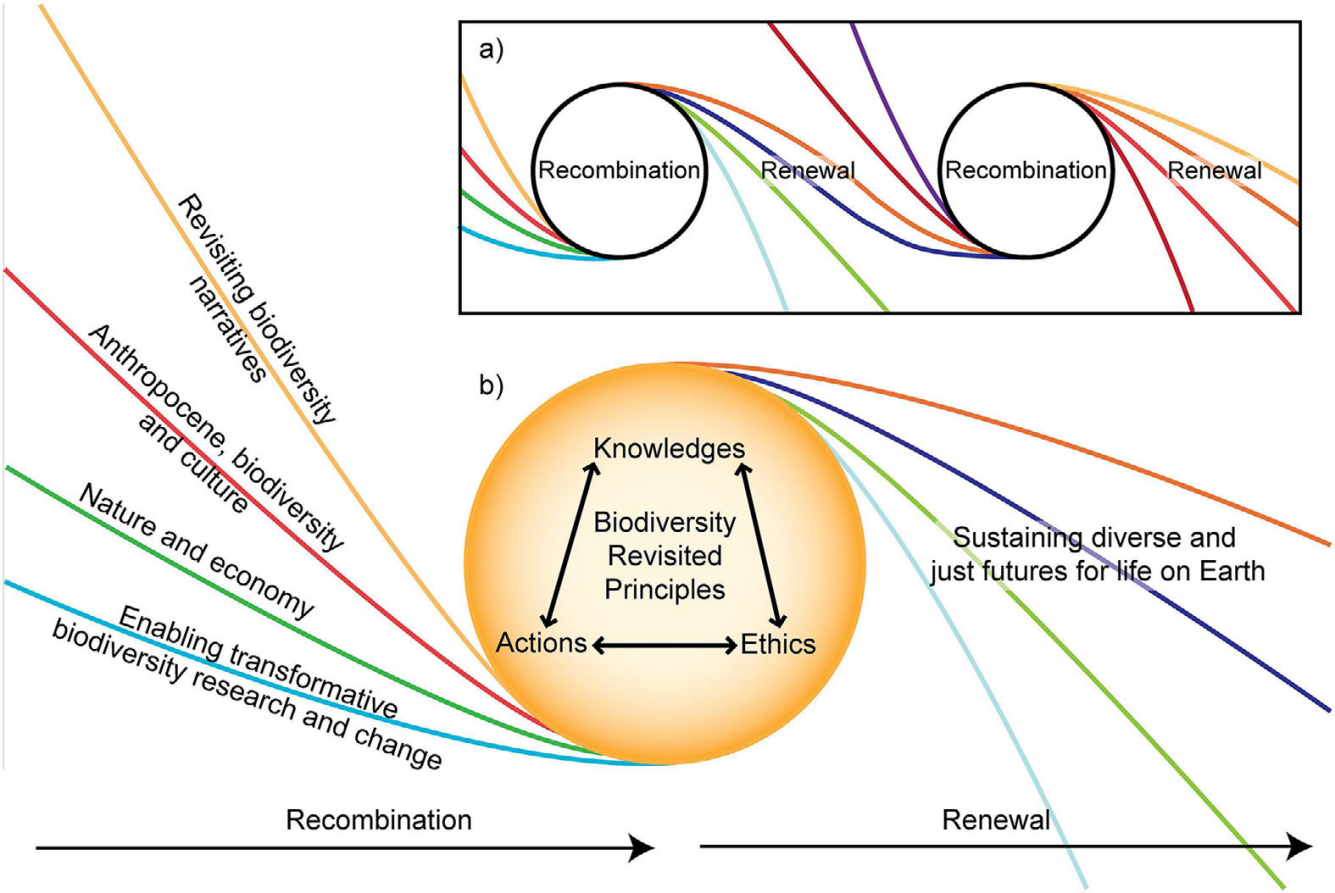
The cyclical process of recombination and renewal of a biodiversity revisited approach (i.e., to critically reflecting on past and present research, policy, and practice concerning biodiversity to think creatively about the future): (a) how the thematic areas of the revisiting biodiversity agenda feed into an ongoing process of research, action, and reflection and (b) how the approach enhances movement toward diverse and just futures for life on Earth.

Revisiting biodiversity starts by recognizing that biodiversity research and action are always in the making and subject to constant evolution. It entails reflecting on past experience, existing concepts, and established practices in an iterative process of recombination and renewal (Fig. 1). Building on the “multiple evidence based approach” (Tengö et al. 2014), recombination weaves together different knowledge to foster “regenerative relationships” (van Kerkhoff 2014) through iterative and interconnected collaborations (i.e., Montana 2019). The process is adaptive and flexible in response to change and is relevant to diverse knowledge systems, including the biophysical sciences, social science, humanities, indigenous, local, and experiential knowledge. This approach acknowledges that working with diverse perspectives toward the goal of this agenda may not require uniformity, convergence, or integration.

**Figure 2:**
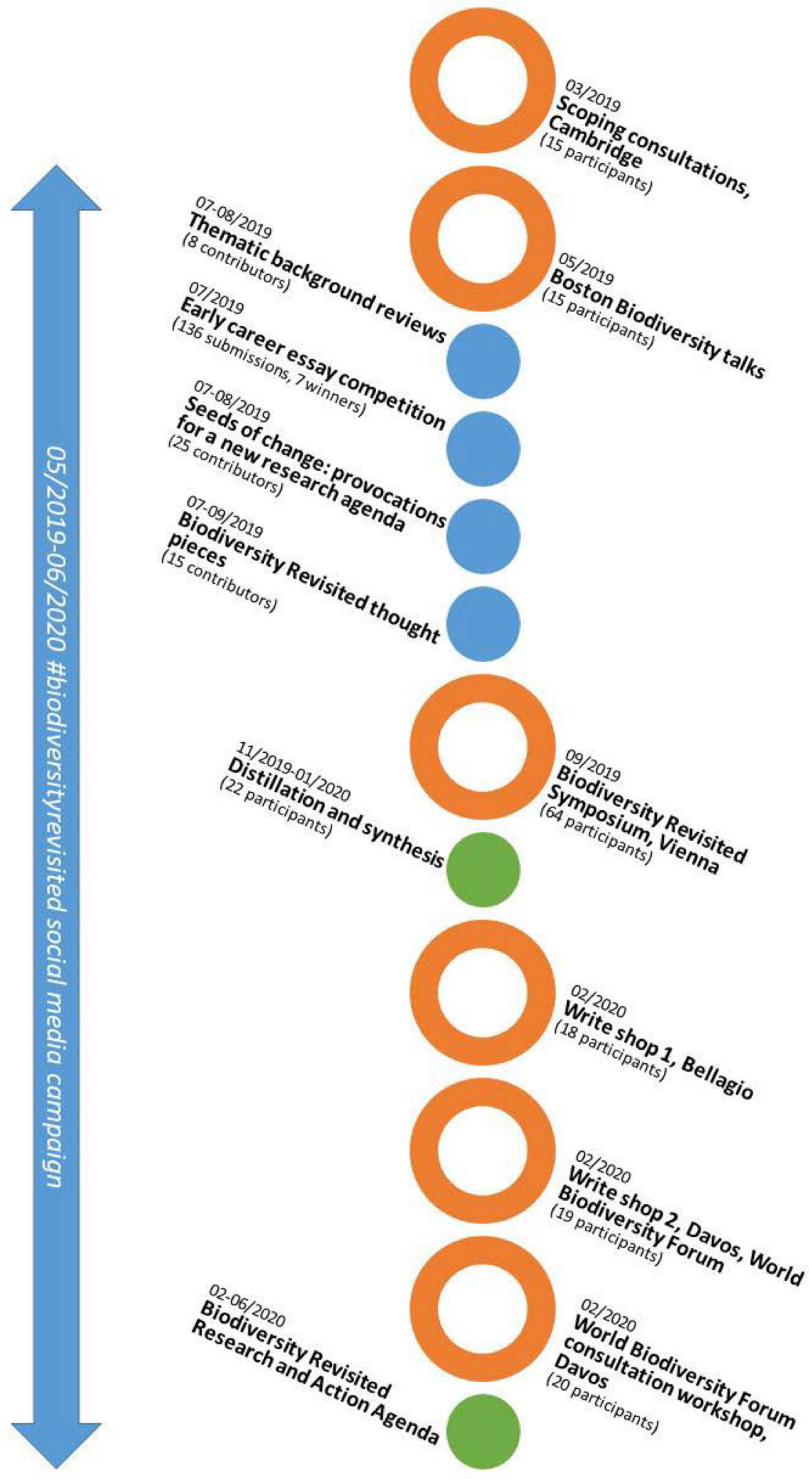
Timeline of the Biodiversity Revisited Initiative from February 2019 to Jun 2020 (orange circles, in‐person meetings; blue circles, written inputs to the process; green circles, series of online meetings and discussions). The process involved close to 300 people in total, but there was a core group of 5–10 people coleading the process. The number of participants on the timeline is a total by event.

This iterative approach was piloted and refined through the Biodiversity Revisited Initiative. The process involved 6 multiday reflective meetings both virtual and in person supplemented by written inputs (Fig. 2). Written inputs provided a starting point for the flagship event, the Biodiversity Revisited Symposium, where a dialogue process was used iteratively and qualitatively to refine the themes for the agenda (Table 1 & Appendix S1). The process was guided by an explicit intention not to reach consensus. Based on the assumption that diversity is key to furthering biodiversity research (Tallis & Lubchenco 2014; Burgman et al. 2015; Mammides et al. 2016), the process welcomed a plurality of perspectives and intentionally allowed for debate and tension (Hulme et al. 2020). In accordance with the ethos of this agenda, the Biodiversity Revisited Initiative was just a small step toward the more ambitious transformative potential of revisiting biodiversity. Ongoing efforts necessitate greater effort to overcome limitations of geographic and epistemic diversity, citation biases, and the exclusion of marginalized voices that lack access to the privileged spaces of such an initiative. Future iterations must start by embracing the project of decolonizing research and adopting an ethic of incommensurability (Tuck & Yang 2012) to address the structural and systemic challenges that perpetuate a Global North bias in biodiversity research and action (Burgman et al. 2015; Nagendra 2018).

**Table 1:** Iterative development of themes related to the revisiting biodiversity agenda

Initial project themes	Themes discussed at Biodiversity Revisited Symposium	Themes in the revisiting biodiversity agenda to sustain diverse and just futures for life on Earth
biodiversity concepts biodiversity narratives biodiversity science biodiversity governance Systems approaches biodiversity futures	justice, accountability, rights and equal representation biodiversity and intergovernmental processes transformative change climate and biodiversity economy, capital, nature Anthropocene and biodiversity knowledge, identities, and biodiversity new conservation ethic and practice individual change? institutional change? systems change? funding, structures and mechanisms of doing research scaling? coexistence and competition poverty, inequality, and colonialism politics, democracy, and pluralism	revisiting biodiversity narratives Anthropocene, biodiversity, and culture nature and economy enabling transformative biodiversity research and change

The Biodiversity Revisited Initiative was guided by 9 principles that were iteratively refined throughout the process (Table 2). Principle‐based approaches that emphasize ethical dimensions are increasingly recognized as important for socioecological research and action (i.e., CBD 2004; van Kerkhoff 2014). Here, knowledge, actions, and ethics are inherently interconnected and mutually sustaining components that structure human relations with the biosphere (e.g., Jasanoff 2004). Knowledge does not just tell one about the world, it actively shapes how one acts within it (Turnhout et al. 2016). Although the principles are not concrete steps for action, we found that recognizing and reflecting on the connections between knowledge, actions, and ethics through these principles provided a means for guiding decision making throughout the initiative. Similar forms of reflexivity will be needed for those who adopt this agenda (Montana et al. 2020). We invite readers to take forward these principles and approach to revisiting biodiversity as a framework for querying their own decisions and actions when funding or contributing to the ongoing agenda.

**Table 2:** Principles underpinning biodiversity revisited research and action

Principle	Definition
Pluralism	Principle recognizes there are multiple ways of knowing, doing, and valuing life on Earth. Pluralism emphasizes the benefit that comes from this diversity of thought rather than forcing consensus or privileging dominant approaches (e.g., Colloff et al. 2017; Díaz‐Reviriego et al. 2019; IPBES 2019).
Reflexivity	Principle emphasizes the value of being open‐minded and aware of one's own assumptions and biases to engage in ongoing learning and improvement. Reflexivity enables flexibility, adaptation, and innovation, and if required transformation, in the face of change (e.g., Pereira et al. 2020).
Humility	Principle compels one to listen to others, as well as speak, and to consider the ethical implications of our actions. Humility is vital in urgent and uncertain times and can cultivate an awareness of the limitations of one's knowledge and actions in a globally connected and complex world (e.g., Pianalto 2013).
Adaptivity	Principle acknowledges that change is constant, unexpected, and often contested. Adaptability harnesses the ability to respond to changing conditions, perspectives, and knowledge as they are encountered (e.g., IUCN 2016; Colloff et al. 2017).
Pragmatism	Principle recognizes the need to work for common benefits in the face of uncertainty. Pragmatism emphasizes the value of gaining knowledge through practical experience while engaging in conscious reflection on existing knowledge, habits, and beliefs (e.g., Robinson 2011).
Inclusivity	Principle fosters meaningful participation of new or previously unacknowledged or underrepresented voices. Inclusivity values diverse contributions to change, and shared leadership in sustained and equitable outcomes (e.g., Tallis & Lubchenco 2014; Díaz‐Reviriego et al. 2019).
Fairness	Principle fosters a trusted and transparent system of allocation. Fairness engenders solidarity with and response‐ability toward the diversity of human and nonhuman life on Earth now and into the future. This requires one to actively work against sources of injustice in research and practice (CBD 2004; Borrini‐Feyerabend & Hill 2015).
Innovativeness	Principle embraces creativity and experimentation and removes unnecessary barriers to exchanging and developing new ideas. Innovation recognizes learning beyond academic institutions to facilitate open‐source solutions and knowledge exchange (e.g., Borrini‐Feyerabend & Hill 2015).
Accountability	Principle denotes explicit and open responsibility for the intended and unintended implications throughout the process of research and practice. Accountability emphasizes the need for a shared liability and commitment (Borrini‐Feyerabend & Hill 2015).

## Thematic Focal Areas

The dialogue process identified 4 themes and priorities for research and action over the next 5 years that contribute to the agenda's goal and catalyze broad engagement in the ongoing task of revisiting biodiversity. The themes and priorities are not exhaustive. These themes, and others, can be further developed following the iterative process of recombination and renewal. Each offers indicative questions that could inform transdisciplinary research on both the social–ecological dynamics and implications of change.

## Revisiting Biodiversity Narratives

Narratives analysis can identify the values, histories, knowledge systems, and worldviews that shape how human–nature relationships are perceived and offer insight into how biodiversity research and action could become more diverse, effective, and just. Narratives can be powerful, emotive stories that incentivize collective action (Rose 2018). Narratives are not neutral descriptions of reality: they frame issues, determine which actors are included or excluded, define cause and effect, assign culpability, and prescribe action (Stone 1989). Once entrenched, dominant narratives can be hard to supplant, even in the face of contradictory evidence (Roe & Eeten 2004). In revisiting biodiversity narratives, we identified 3 areas toward which research could productively focus.

### Bringing Diverse Perspectives and Approaches to Narratives Together to Enrich Biodiversity Research

Focusing on narratives can enable “unprecedented listening” by questioning which knowledge sources hold authority and what other knowledge and options these close down (Veland et al. 2018). Narrative analysis can facilitate productive dialogue among knowledge systems, including indigenous and local knowledge systems, and disciplines across the arts, humanities, psychology, and cognitive science. The very individuals, communities, and people who are needed to diversify narratives too often have little opportunity to engage in privileged research processes. Widening participation can acknowledge histories of colonization that have erased biodiverse knowledge in order to address limited practices of consultation and exchange. Researchers could examine how those involved in biodiversity‐related work can more effectively listen to and learn from narratives that have been traditionally outside of biodiversity research.

### Empirically Examining Narratives that Underpin Destructive Systems

Analysis of narratives can provide insight into underlying factors shaping human–nature relationships (Veland et al. 2018). There is an evident need to address structural racism and geographic biases within biodiversity research and practice more broadly (Burgman et al. 2015; Editors Nature Ecology & Evolution 2020). Such analyses can unpack the narratives that perpetuate unjust and unsustainable outcomes, by focusing on the distribution of costs and benefits of actions, and make explicit the power relations that may be naturalized in narrative. Future researchers could examine what makes dominant narratives authoritative and stable (Roe & Eeten 2004) by studying, for example, why some narratives become authoritative and unquestioned, whereas others are silenced or deliberately ignored, and what the results of such narratives are.

### Exploring the Role of Narratives in Imagining Alternative Futures and Enabling Transformative Change

Research on climate narratives shows how local narratives may catalyze more meaningful action than those adopting ideas of causality and solutions based on physical science representations (Krauß 2020). Narratives therefore provide an important foundation for creative and emotive ways of imagining the future. Researchers increasingly call for participatory processes to envision radically different and positive futures to overcome the limitations of technocratic approaches in motivating action (Veland et al. 2018; Pereira et al. 2020) and could examine how narratives and narrative approaches can be used to foster productive engagement with contested and uncertain futures.

## Anthropocene, Biodiversity, and Culture

This theme builds on research noting the potential of the Anthropocene concept (Arias‐Maldonado 2020), to suggest that revisiting biodiversity necessitates greater attention to contextually appropriate and community‐led innovations that accommodate diverse cultures and knowledge systems. Earth system science largely focuses on the novelty of pace, scale, and complexity of human impacts on the planet in the Anthropocene and has informed research in the biodiversity and the sustainability science communities (i.e., Steffen et al. 2015). However, the transformative potential of the Anthropocene concept is limited when it simplifies complex change processes into a uniform narrative of a destructive humanity that does not consider diversity, equity, responsibility, and the economic drivers of social–ecological degradation (Dalby 2016). For example, growing evidence globally emphasizes the contribution of Indigenous peoples, knowledge systems, and practices in maintaining biodiverse ecosystems (Roe & Eeten 2004) through longstanding cultural and spiritual connections to their land and seascapes (Garnett et al. 2018). Yet, the value of culture in biodiversity conservation is underexplored. In revisiting biodiversity through this theme, we identified 4 core areas.

### Cultivating Deeper Understanding of Interconnected Social–Ecological Systems

The majority of today's landscapes, cultures, and biodiversity coevolved through place‐based interactions between humans and nonhuman species (Rozzi et al. 2018). This diversity is intimately linked (linguistically, culturally, biologically) and mutually sustaining (Gorenflo et al. 2012). We call for continued research that examines the world's social–ecological systems (their origins, composition, functions, and dynamics) to address the following question: What physical, psychological, and philosophical connections and conditions are important to shaping knowledge, actions, and ethics about nature in different places?

### Reconsidering Human Agency, Accountability, and Responsibility in Shaping the Anthropocene

The importance of culture and history in conservation is underappreciated. Participation, resource distribution, and cultural recognition matter to biodiversity research and action and raise important questions about justice (Martin et al. 2016). Research is therefore needed to explore the role of human agency in navigating the challenges of the Anthropocene and alternative mechanisms of governance that can enable accountability and responsibility for problems where cause and effect are distributed across time and space (Burch et al. 2019). This research needs to account for variation in historical, present, and future accountability and responsibility by examining what governance actors and processes can most appropriately tackle the fundamental challenges of the Anthropocene.

### Developing Solutions that Embrace Context‐Based Knowledge and Multiple Values

Research contributions should account for the loss of biological and cultural diversity as land use, diets, and biotic communities become homogenized (Khoury et al. 2014; Nyström et al. 2019). At its core, this research could recognize uncertainty due to a lack of analogous historic states as central to the Anthropocene. Research should examine the appropriateness of conservation interventions when things are no longer considered “stable, pristine and certain” (Head 2018) and human values more explicitly underpin justifications for action. Research is needed to identify solutions that embrace appropriate context‐based knowledge and multiple values by considering what mechanisms of change (across scales and contexts) can lead to more just, prosperous, and ecologically diverse futures and who decides.

### Balancing the Needs for Context‐Driven Responses to Widespread Global Challenges

Local biodiversity and culture are affected by globally interconnected social, economic, and ecological drivers (e.g., telecoupling [Liu et al. 2016]). The Anthropocene presents a paradox: the challenges are global, but effective solutions require smaller scale, context‐specific interventions. Recognizing this tension, we invite researchers to examine what modes of social and political organization might balance contextualized concerns that promote and support difference and desires for cooperation and coordinated responses that span sites and scales.

## Nature and Economy

Revisiting biodiversity in this theme involves challenging existing economic models, exploring new financial responses to the biodiversity crisis, and catalyzing innovative ways of understanding and transforming global social–ecological systems. Economic paradigms that separate nature and biodiversity from social and economic systems have fostered a dominant way of valuing and relating to nature as a resource or capital for human production, consumption, or exchange. The resulting patterns of production, trade, finance, and consumption drive biodiversity loss, economic degradation, and commonly prioritize particular interests over collective well‐being, which perpetuates social inequalities (IPBES 2019). Addressing the degradation of biodiversity includes transforming global economic systems alongside underlying narratives about how humans, economies, and biodiversity relate and depend on each other. We do not conceptualize nature, capital, and economy as existing in an absolute sense; instead, we use these terms to anchor discourse to promote particular relations between nature and society (Escobar 1998). For example, common definitions of biodiversity and nature denote discrete scientific phenomena separate from humans and the economy, which are seen as supported through stocks of accumulated capital. Although widely used, these definitions promote extractive and competitive relations and logics that can inhibit transformation. We call for the acknowledgement of the performativity of definitions and the need to openly explore alternatives that define nature and the economy as fundamentally interdependent (Moore et al. 2014) through 3 core areas of focus.

### Challenging Business as Usual

Existing economic paradigms and models largely frame nature and economy as separate, supporting efficient resource use and economic growth rather than absolute reductions in consumption and ecological impacts (Otero et al. 2020). Despite growing evidence of negative social–ecological effects of dominant economic practices (IPBES 2019), political and practical change has proven difficult, particularly with respect to decoupling economic growth from biodiversity loss and inequality (Otero et al. 2020). To better understand the processes and powers that reinforce this separation in policy and practice, research is needed to investigate what factors underlie current economic paradigms and practices, how these factors reinforce a separation of nature–economy relations, and how these factors can be reshaped.

### Exploring Incremental Change

Despite growth in initiatives seeking to account for biodiversity in market logics (e.g., Natural Capital, Payments for Ecosystem Services), these approaches often fail to achieve desired conservation or social outcomes at scale as they are not embedded in an enabling regulatory and economic environment and do not challenge the status quo (McAfee 1999; Hein et al. 2020). Research is needed to examine the impact of incremental approaches (discrete measures aimed at adjusting a given course of action) by improving methods to monitor and understand their efficacy from a long‐term and integrated perspective, including investigating distribution of costs and benefits, leakages, substitutions, and impacts across scales. Research is needed to examine how incremental efforts can support (rather than inhibit) transformative efforts toward just, equitable, and sustainable nature–economy relations.

### Catalyzing Fundamental Change

The entrenched logic of the predominant global economic paradigm makes it difficult for research alone to destabilize the mental models, ideologies, assumptions, and practices underpinning the economic drivers affecting biodiversity. Radical initiatives are emerging to reshape the global economic system to value nature using alternative and pluralistic narratives (e.g., postconsumerism, economies of sufficiency, degrowth, universal basic services, nature's contributions to people) (Portes et al. 2017; Raworth 2017; IPBES 2019). Such innovation has potential to create economic systems that are more resilient and conducive to environmental integrity and social justice. Research should examine how diverse approaches to transforming economies can be harnessed to counter dominant economic logics and nature–economy relations; how research can engage diverse actors in joint efforts to understand and reshape nature–economy relations; and what the risks and ethical implications of such engagements are.

## Enabling Transformative Biodiversity Research and Action

This theme focuses on the ways individuals and institutions can enable transformative change in the ways people understand, value, and relate to human and nonhuman forms of life through embracing plural knowledge, values, and cultures. Transformative change is likely to involve major shifts in the underlying paradigms and values that shape technologies, governance, economies, and nature (IPBES 2019). Transformation is never apolitical: it requires careful scrutiny about whom transformations are for, what is to be transformed, and how these things are to be decided (Blythe et al. 2018; Scoones et al. 2020; Pereira et al. 2020). The burgeoning literature on transformative change provides critical insights for transformations‐oriented work for biodiversity (e.g., Westley et al. 2011; O'Brien 2012). Some headway has been made in discussing how to transform conservation science for the Anthropocene (Colloff et al. 2017). Directing this work toward revisiting biodiversity, we identified 4 priority areas.

### Learning from Past Transformations

Agency and transformability of individuals and institutions are key to implementing structural, systemic, and enabling approaches to transformation (Scoones et al. 2020). We call for research that critically evaluates these broad approaches to transformations to identify common elements of previous transformations so as to understand and unpack the current and future transformations by examining how previous transformative changes to biodiversity occurred and how understanding these past transformations can help in planning for the future.

### Institutional and Individual Roles in Transformative Change

Individuals and institutional capacities to enact transformative change are codependent and guided by their underlying ethics, paradigms, and discourses. Transformative change entails rethinking training for researchers and policy professionals and the ideas that are currently privileged by conservation agendas. This includes addressing structural racism and geographic biases within biodiversity research and publishing (Burgman et al. 2015; Editors Nature Ecology & Evolution 2020), as well as the methodologies (Chilisa 2017) and Western academic structures (Nyamnjoh 2019) that devalue stories about nature relationships from diverse parts of the world (Nagendra 2018). Decoloniality is a long‐term project that requires commitment to generations of scholars, practitioners, and knowledge holders to acknowledge past injustices and open up spaces for more active contributions from a fully diverse group (Tuck & Yang 2012). A more robust understanding of the interplay between individual and institutional change can enhance the transformative potential of biodiversity research (Moore et al. 2014). This leads us to pose the reflexive question: How is biodiversity research contributing to understanding, or enabling, transformative change toward diverse, sustainable, and just futures?

### Inclusive and Plural Transformations

Conducting transformative research requires changes to how institutions fund, conduct, and value research and action. Despite increased calls for interdisciplinarity and incorporation of non‐Western knowledge systems, traditional funding mechanisms tend to focus on research that is tightly bound to a singular disciplinary focus with clearly defined objectives and outcomes (Hakkarainen et al. 2020). More work is needed to examine what approaches to research and action can catalyze or block transformation, how to foster pluralism and diversity, and in particular how to make marginalized voices and scholarship integral to transformative biodiversity research (Tengö et al. 2014; Latulippe & Klenk 2020). This work could start with identifying practical means through which to rectify the structural inequalities and the extractive traditions of knowledge production that underpin (biodiversity) research (Editors Nature Ecology & Evolution 2020). This requires knowing what tools, narratives, and approaches are needed to embrace a plurality in perspectives on what transformations should occur and to provide pathways for multiple futures that can coexist.

### Research and Action in Light of Uncertainty

Transformative change processes are inherently uncertain because it is very difficult to know whether an event is transformative and how a system will respond (Blythe et al. 2018; Pereira et al. 2020). New capacities for transformative thinking and learning are required to anticipate change and conceptualize alternative futures, so as to enable informed decisions in the present while acknowledging inherent uncertainties of the future (Vervoort & Gupta 2018). Researchers should examine how to build capacities to anticipate transformations and still take action despite uncertainties regarding how social–ecological systems respond to change.

## Toward Sustaining Diverse and Just Futures for Life on Earth

The ongoing task of revisiting biodiversity will take many forms. This agenda is intended as an initial resource that offers a renewed vision of the what and the how of future transdisciplinary research and action for biodiversity and social justice. Of course, social–ecological issues have messy realities; can be conceptualized and researched in many ways; and might lead to an array of desirable futures. As such, this agenda seeks to inspire, rather than prescribe, collaborative engagement between different sectors of society and academia. Privileging particular actions or strategies has consequences for who is empowered or marginalized, which forms of knowledge are legitimized, and what issues receive attention. The search for silver bullets in biodiversity research and action has created perverse social and ecological outcomes and perpetuated social inequalities. Moving away from the universalist and global tendencies that plague biodiversity research and action (Turnhout et al. 2016), this agenda is put forward with humility to be renegotiated and revised within localized contexts and concerns, where tangible actions are critical to affect change. We encourage projects, institutions, and research endeavors to identify appropriate actions by engaging with, and critically reflecting on, diverse perspectives, visions, and stakes to consider the costs, benefits, and implications of future biodiversity research and action. Ultimately, new directions of many kinds are needed to foster more integrated, inclusive, and transformative approaches to biodiversity research and action that will enable more diverse and just futures for life on Earth.

## Supporting information

Additional information is available online in the Supporting Information section at the end of the online article. The authors are solely responsible for the content and functionality of these materials. Queries (other than absence of the material) should be directed to the corresponding author.

Supplementary Material
