# Supplementary material for: An agenda for research and action toward diverse and just futures for life on Earth
Source: Conserv Biol. 2021 Mar 3;35(4):1086–97. doi: 10.1111/cobi.13671 (PMC8359367; doi:10.1111/cobi.13671)
Supplement: Supplementary file 1 — Additional information is available online in the Supporting Information section at the end of the online article. The authors are solely responsible for the content and functionality of these materials. Queries (other than absence of the material) should be directed to the corresponding author. [file COBI-35-1086-s001.docx]

# Supplementary Material: Biodiversity Revisited Symposium.

The Symposium was held in Vienna, September 2019, and attended by 64 experts from 29 nations. Members of the Initiative’s Steering Committee initially nominated experts (see CITATION REMOVED FOR PEER REVIEW), defined as *a person with an intimate understanding of a subject matter, derived from either abstract/generalizable knowledge (for example gained through formal academic training) or local/practical knowledge (for example, gained through experience, tradition or practice)* (adapted from Carolan 2006). Symposium participants were then identified through a purposive and snowball sampling method (Bryman 2015). The Initiative did not aspire to be fully representative of all disciplines and perspectives as this is not practicable at the global level. Instead, six diversity metrics were identified to select the participants - gender, career stage, discipline, sector, region of employment, and nationality by region. They were broadly balanced across gender and career stages, and interdisciplinary expertise (Table 1). Despite significant effort to include participants employed outside North America and Europe, including funding for travel expenses, these remained the dominant regions. Any future work needs to move beyond this limited constituency to build more inclusive participation, particularly from the Global South.

**Table 1. Breakdown of Symposium participants and authors by multiple diversity metrics**

| Diversity metrics | Symposium participants n=64 | Agenda^b^ authors n=22 |
| --- | --- | --- |
| Gender | M - 33  F - 30  NB - 1 | M - 6  F - 16 |
| Career stage^c^ | Senior - 24  Mid career - 25  Early career - 15 | Senior - 5  Mid career - 7  Early career - 10 |
| Discipline | Natural science - 16  Social science - 19  Interdisciplinary - 24  Humanities - 3  Communication - 2 | Natural science - 1  Social science - 6  Interdisciplinary - 13  Humanities - 2 |
| Sector | Academia - 35  NGO - 14  Government - 4  Intergovernmental - 2  Funder - 2  Publishing - 1  Private sector - 1  Think tank - 1  Independent - 1 | Academia - 18  NGO - 3  Private sector - 1 |
| Continent of employment | Australasia - 7  Europe - 23  North America - 13  Latin America - 7  Africa - 7  Asia - 7 | Australasia - 3  Europe - 7  North America - 7  Latin America - 3  Africa - 1  Asia - 1 |
| Nationality by region | Australasia - 6  Europe - 17  North America - 12  Latin America - 9  Africa - 11  Asia - 9 | Australasia - 4  Europe - 6  North America - 5  Latin America - 4  Africa - 2  Asia - 1 |

Abbreviations: n: number of people; M: male; F: female; NB: non-binary.

^a^ The Biodiversity Revisited Symposium was held in Vienna, Austria, 11-13 September 2019.

^b^ Researchers and practitioners who developed the Biodiversity Revisited Research and action agenda for sustaining diverse and just futures for life on Earth (Citation removed for peer review)

^c^ Career stages: Senior: professor or senior practitioner; Mid-career: established researcher or practitioner; Early career: researcher or practitioner maximum five years post final qualification.
