# Supplementary material for: An agenda for research and action toward diverse and just futures for life on Earth
Source: Conserv Biol. 2021 Mar 3;35(4):1086–97. doi: 10.1111/cobi.13671 (PMC8359367; doi:10.1111/cobi.13671)
Supplement: Supplementary file 2 — Supplementary Material [file COBI-35-1086-s002.pptx]

## Slide 1
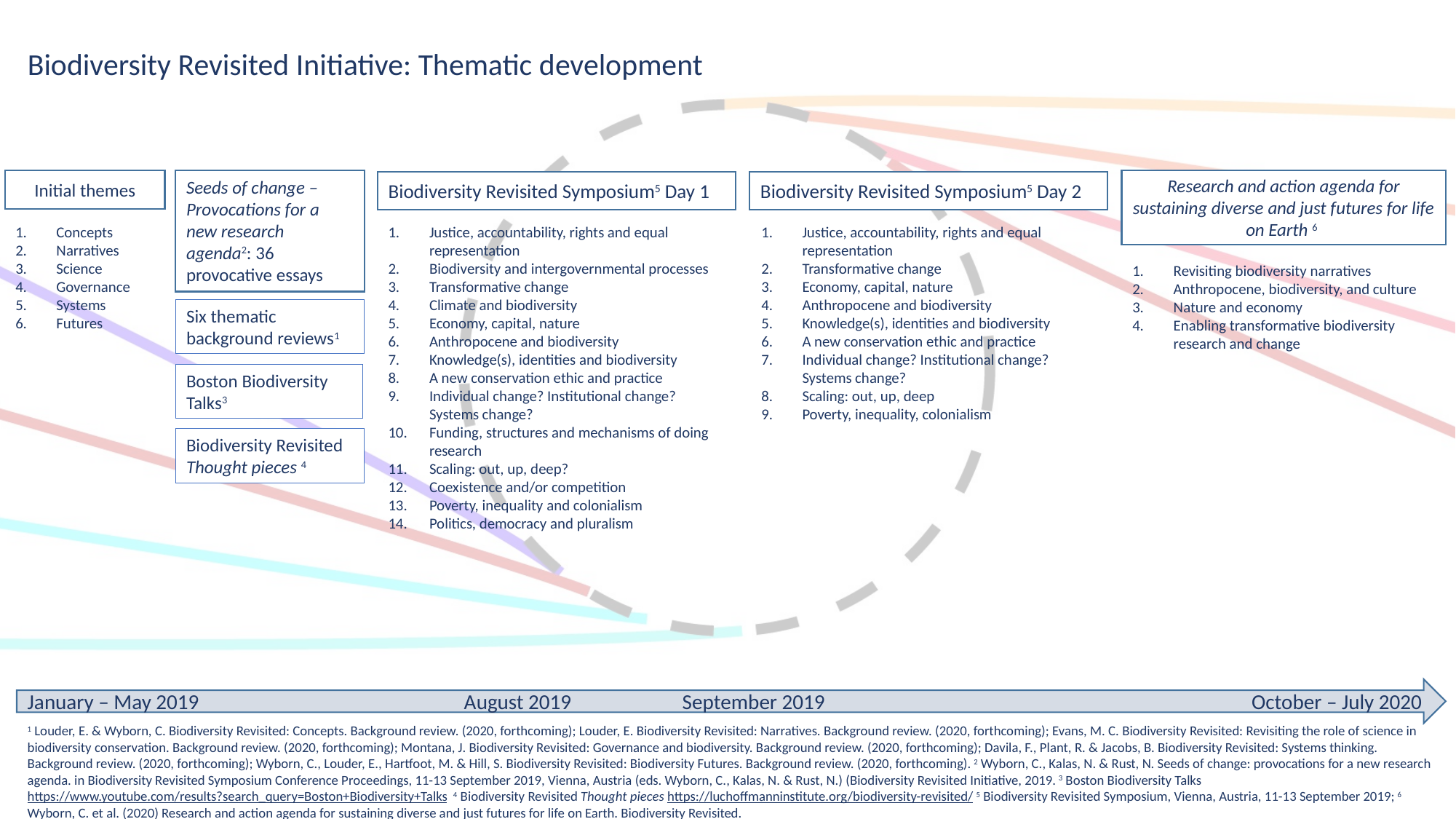

Biodiversity Revisited Initiative: Thematic development
Seeds of change – Provocations for a new research agenda2: 36 provocative essays
Initial themes
Research and action agenda for sustaining diverse and just futures for life on Earth 6
Biodiversity Revisited Symposium5 Day 1
Biodiversity Revisited Symposium5 Day 2
Concepts
Narratives
Science
Governance
Systems
Futures
Justice, accountability, rights and equal representation
Biodiversity and intergovernmental processes
Transformative change
Climate and biodiversity
Economy, capital, nature
Anthropocene and biodiversity
Knowledge(s), identities and biodiversity
A new conservation ethic and practice
Individual change? Institutional change? Systems change?
Funding, structures and mechanisms of doing research
Scaling: out, up, deep?
Coexistence and/or competition
Poverty, inequality and colonialism
Politics, democracy and pluralism
Justice, accountability, rights and equal representation
Transformative change
Economy, capital, nature
Anthropocene and biodiversity
Knowledge(s), identities and biodiversity
A new conservation ethic and practice
Individual change? Institutional change? Systems change?
Scaling: out, up, deep
Poverty, inequality, colonialism
Revisiting biodiversity narratives
Anthropocene, biodiversity, and culture
Nature and economy
Enabling transformative biodiversity research and change
Six thematic background reviews1
Boston Biodiversity Talks3
Biodiversity Revisited Thought pieces 4
January – May 2019			August 2019		September 2019				 October – July 2020
1 Louder, E. & Wyborn, C. Biodiversity Revisited: Concepts. Background review. (2020, forthcoming); Louder, E. Biodiversity Revisited: Narratives. Background review. (2020, forthcoming); Evans, M. C. Biodiversity Revisited: Revisiting the role of science in biodiversity conservation. Background review. (2020, forthcoming); Montana, J. Biodiversity Revisited: Governance and biodiversity. Background review. (2020, forthcoming); Davila, F., Plant, R. & Jacobs, B. Biodiversity Revisited: Systems thinking. Background review. (2020, forthcoming); Wyborn, C., Louder, E., Hartfoot, M. & Hill, S. Biodiversity Revisited: Biodiversity Futures. Background review. (2020, forthcoming). 2 Wyborn, C., Kalas, N. & Rust, N. Seeds of change: provocations for a new research agenda. in Biodiversity Revisited Symposium Conference Proceedings, 11-13 September 2019, Vienna, Austria (eds. Wyborn, C., Kalas, N. & Rust, N.) (Biodiversity Revisited Initiative, 2019. 3 Boston Biodiversity Talks https://www.youtube.com/results?search_query=Boston+Biodiversity+Talks 4 Biodiversity Revisited Thought pieces https://luchoffmanninstitute.org/biodiversity-revisited/ 5 Biodiversity Revisited Symposium, Vienna, Austria, 11-13 September 2019; 6 Wyborn, C. et al. (2020) Research and action agenda for sustaining diverse and just futures for life on Earth. Biodiversity Revisited.
